# Impact of the discrepancy between predicted and actual ring size on the outcomes of mitral valve repair using the loop technique

**DOI:** 10.1016/j.xjon.2025.07.008

**Published:** 2025-07-21

**Authors:** Koji Yamana, Yoshiyuki Takami, Wakana Niwa, Kazuki Matsuhashi, Kentaro Amano, Kiyotoshi Akita, Atsuo Maekawa, Yasushi Takagi

**Affiliations:** Department of Cardiovascular Surgery, Fujita Health University, Toyoake City, Aichi, Japan

**Keywords:** size discrepancy of annuloplasty ring, mitral valve repair, loop technique, coaptation length

## Abstract

**Objective:**

To investigate the impact of the discrepancy between the predicted annuloplasty ring size determined by preoperative transesophageal echocardiography and the actual ring size on the outcomes of mitral valve repair (MVr) using primarily the loop technique.

**Methods:**

Among 370 patients who underwent MVr between January 2008 and December 2024, 154 who underwent MVr with the semirigid ring for degenerative disease were involved in this study. Ring size was estimated according to the lengths of A2 and P2 by transesophageal echocardiography. Patients were classified into 3 groups—small, match, and large—based on the discrepancy between the predicted and actual ring sizes.

**Results:**

Compared with the other small and match groups, patients in the large group had shorter anterior leaflet length (*P* = .03), smaller posterior leaflet angle (*P* = .01) and smaller coaptation depth (*P* = .03) in the coaptation triangle. There was no significant difference in coaptation length among the 3 groups. The cumulative incidence of mitral regurgitation (MR) grade ≥2 was 0.6% at 1 year, 5% at 5 years, and 24% at 10 years, whereas that of a mean transmitral pressure gradient ≥5 mm Hg were 5%, 13%, and 16%, respectively. Fine-Grey multivariable analysis identified larger posterior leaflet angle after repair as a risk factor for recurrence of MR grade ≥2 and larger body surface area, smaller prosthesis size, and shorter coaptation length as risk factors for functional mitral stenosis.

**Conclusions:**

Size mismatch of the prosthesis ring did not change the coaptation length, late MR recurrence, or functional mitral stenosis after MVr.

**Figure:**
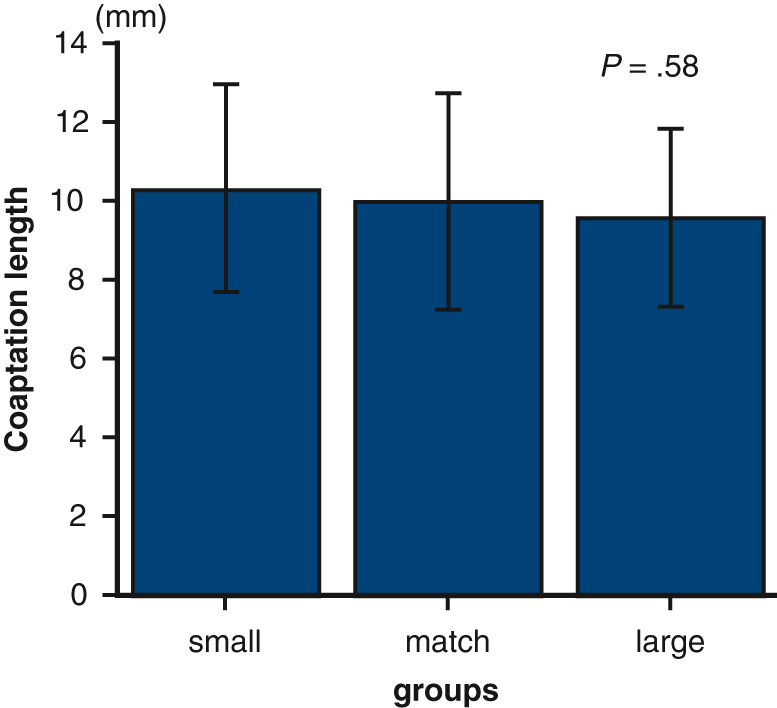
Discrepancy in ring size in mitral valve repair with the loop technique did not alter coaptation length.

**Central Message**

In mitral valve repair using the loop technique, no significant difference in coaptation length was observed irrespective of the magnitude of the discrepancy between predicted and actual ring sizes.

**Perspective**

In mitral valve repair using the loop technique, discrepancies in ring size did not affect coaptation length. Consequently, the loop technique enables implantation of larger rings, potentially ensuring adequate central coaptation of the mitral valve to avoid recurrence of mitral regurgitation and prevent long-term functional mitral stenosis.

Implantation of a mitral annuloplasty ring is an essential part of mitral valve repair (MVr) to restore the size and shape of the native annulus and stabilize it for long-term durability.^1^, ^2^, ^3^, ^4^ When selecting the appropriate ring size, including a sufficient amount of leaflet tissue in coaptation is considered especially critical.^5^, ^6^, ^7^ Inadequate annuloplasty ring size may lead to an unfavorable repair, either with residual mitral regurgitation (MR) if the ring size is too large or with functional mitral stenosis (MS) or systolic anterior motion (SAM) if the selected ring is too small.^7^, ^8^, ^9^ Mitral annuloplasty ring sizing usually is performed using a commercial sizer model intraoperatively during cardiac arrest on the unloaded and nonbeating heart. Sizing is based on the intertrigonal distance or the height of the anterior leaflet,^8^ which differs from that of the beating and loaded heart.

At our center. transesophageal echocardiography (TEE) is performed preoperatively to accurately identify the etiology, lesion, and extent of the mitral valve (MV) disease. TEE makes it possible to more accurately measure the height of the anterior and posterior leaflets on the loaded and beating heart, enabling prediction of the appropriate annuloplasty ring size that may obtain sufficient coaptation and is not too small. This study was conducted to investigate the impact of the discrepancy between the predicted annuloplasty ring size determined using preoperative TEE and the actual implanted ring size on the morphology of the MV after repair, particularly the depth of coaptation, residual MR, and occurrence of functional MS and SAM. We also investigated the influence on the recurrence of MR and the development of functional MS in the mid-term and long-term follow up.

## Methods

### Ethical Statement

This study was approved by the Fujita Health University Institutional Review Board (reference HM22-417; approved March 8, 2023). The need for informed patient consent was waived.

### Study Cohort and Data Collection

The institutional surgical database contained a consecutive series of 370 patients who underwent primary MVr at Fujita Health University Hospital between January 2008 and December 2024. A total of 94 patients diagnosed with functional type I or III of MR were excluded, leaving a cohort of 276 patients with degenerative MR, of whom 154 underwent MVr with annuloplasty using the semirigid ring enrolled in the present study. A total of 122 patients were excluded from the analysis, including 78 in whom a ring or band other than the semirigid ring were used, 9 in whom no ring or band was used, 21 who did not undergo preoperative TEE, 3 in whom echocardiographic imaging diagnosis was difficult, and 11 in whom artificial chordae were used but without the loop technique. Of the patients who met the inclusion criteria, the patients whose ring size predicted by preoperative TEE matched the actual implanted ring size or had a 1 size up or 1 size down (−1 to +1) ring implanted were categorized as the match group. Those with a ring implanted that was 2 sizes down or more (−2 to −4) were categorized as the small group, and those with a ring implanted that was 2 or more sizes up (+2 to +4) were categorized as the large group. To compare the 3 groups, medical charts, surgical reports, and referral letters were reviewed for data collection. A total of 152 patients (98.7%) completed follow-up, with a median duration of follow-up of 6.4 years (interquartile range [IQR], 4.1-9.0 years).

### Surgical Indications and Techniques

The surgical indications for MVr were discussed by the heart team in accordance with published guidelines.^10^ In all patients, annuloplasty was performed with a Physio II ring (Edwards Lifesciences). The prosthetic size was selected with the sizer instrument intraoperatively based on the height of the mitral anterior leaflet and the intercommissural distance. Although the repair technique depended on surgeon preference, most repairs (88%) were performed with the loop technique^11^ using CV4 polytetrafluoroethylene sutures (WL Gore and Associates). For anterior leaflet prolapse, the loop was sutured to the free margin of the leaflet. When the prolapsed posterior leaflet height was ≥15 mm, it was sutured at a height of 15 mm from the annulus (Video 1). If the posterior leaflet height was ≤15 mm, it was attached to the free edge of the leaflet. The surgical approach was either a standard median sternotomy or a mini right thoracotomy.

### Echocardiography

**Figure 1:**
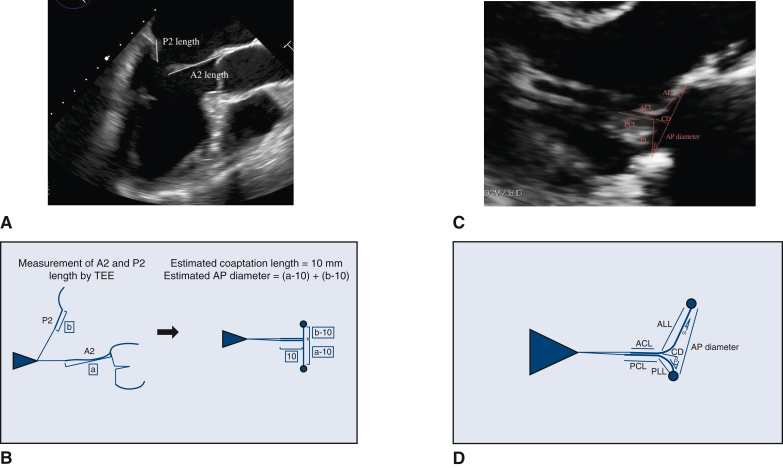
A, The lengths of A2 and P2 measured by preoperative transesophageal echocardiography (TEE) in the midesophageal long-axis view. B, Using an estimated coaptation length of 10 mm, the sum of each leaflet height −10 mm was taken as the estimated AP diameter. C and D, Postoperative echocardiographic measurements included the anterior and posterior leaflet coaptation length, which represent the leaflet length from the coaptation point to the left ventricular side; the coaptation length, which was defined as the shorter of the anterior or posterior coaptation length; the coaptation depth; the anterior and posterior leaflet lengths in the coaptation triangle; and the anterior and posterior leaflet angles relative to the annulus plane. *AP*, Anterior-posterior; *ACL*, anterior leaflet coaptation length; *PCL*, posterior leaflet coaptation length; *AL*, anterior leaflet length; *PL*, posterior leaflet length; *CD*, coaptation depth; α, anterior leaflet angle; β, posterior leaflet angle.

All the patients were examined by standard transthoracic echocardiography (TTE) within 1 month before surgery and again at 2 weeks after surgery in accordance with our institutional clinical practice guidelines. In addition, TEE was also performed within 1 month before surgery for all the patients to investigate the pathophysiology, location, and extent of the lesion of degenerative MV prolapse and the presence of associated lesions in detail. A2 and P2 lengths were measured by TEE, most frequently in the midesophageal long-axis view (Figure 1, *A*). Using a coaptation length of 10 mm, the sum of each leaflet height −10 mm was taken as the anterior-posterior (AP) diameter of the annulus after ring annuloplasty, and the size closest to this was considered the appropriate estimated ring size (Figure 1, *B*).^12^ If the posterior leaflet height was <10 mm, the anterior leaflet height of 10 mm was recorded as the targeted AP diameter. When using the loop technique for P2 prolapse, the posterior leaflet height is 15 mm, so the anterior leaflet height of 10 mm + 5 mm was recorded as the estimated AP diameter. Postoperative TTE measurements included the anterior and posterior coaptation lengths, which represent the leaflet length from the coaptation point to the left ventricular side; the coaptation length, defined as the shorter of the anterior or posterior coaptation length; the coaptation depth; the anterior and posterior leaflet lengths in the coaptation triangle; and the anterior and posterior leaflet angles relative to the annulus plane (Figure 1, *C* and *D*). Follow-up TTE after discharge was performed at 1 year and annually thereafter. MR grade was defined as 0, none or trivial; 1, mild; 2, moderate; 3, moderate to severe; or 4, severe.

### Statistical Analysis

Categorical data are expressed as frequency with corresponding percentage. Continuous data are shown as median (IQR) and checked for a normal distribution using the Shapiro-Wilk test. Intergroup comparisons for categoric data were performed using the χ^2^ test or Fisher exact test. Kaplan-Meier analysis was used to assess mortality, and a competing-risks model was used to assess the incidences of reoperation, MR recurrence, and functional MS occurrence. To assess the independent risk factors for MR recurrence or functional MS occurrence, a multivariable Fine-Gray regression model was used with the following 7 variables: age, body surface area (BSA), chronic atrial fibrillation, prosthesis size, prosthesis size mismatch, postoperative coaptation length, and posterior leaflet angle. These variables were selected based on prior knowledge and clinical experience. There were no missing data for the explanatory variables. A *P* value < .05 was considered to indicate statistical significance. All statistical analyses were performed using EZR statistical software (Saitama Medical Center, Jichi Medical University), a graphical user interface for R (The R Foundation for Statistical Computing).

## Results

### Baseline Characteristics

The baseline characteristics of this study cohort are reported in Table 1. The median patient age was 62 years (IQR, 54-72 years), and the median BSA was 1.65 m^2^ (IQR, 1.5-1.8 m^2^). Left ventricular contraction was well preserved; the median left ventricular ejection fraction was 62% (IQR, 60%-66%). In terms of the location of prolapse, A1, A2, A3 prolapse occurred in 11 patients (7.1%), 38 patients (24.7%), and 26 patients (16.9%), respectively, and P1, P2, and P3 prolapse occurred in 16 (10.4%), 78 (50.6%), and 47 (30.5%), respectively. Commissure prolapse occurred in 22 patients (14.3%); multiple segment prolapse, in 40 patients (26%). Eleven patients (7.1%) were diagnosed with Barlow disease.

**Table 1:** Patient characteristics (N = 154)

Characteristic	Value
Age, y, median (IQR)	62 (54-72)
Male sex, n (%)	107 (69.5)
Body surface area, m^2^, median (IQR)	1.65 (1.51-1.78)
NYHA class ≥3, n (%)	16 (10.4)
Renal dysfunction (Cr > 1.5), n (%)	4 (2.6)
Hypertension, n (%)	94 (61.0)
Hypercholesterolemia, n (%)	44 (28.6)
Diabetes, n (%)	14 (9.1)
Permanent atrial fibrillation, n (%)	43 (27.9)
Paroxysmal atrial fibrillation, n (%)	5 (3.2)
Preoperative echocardiography findings	
LV diastolic diameter, mm, median (IQR)	55 (50-59)
LV systolic diameter, mm, median (IQR)	35 (31-38)
Left atrial diameter, mm, median (IQR)	45 (40-49)
LV ejection fraction, %, median (IQR)	62 (60-66)
TR grade, grade 0-4, median (IQR)	1 (0-1)
Location of prolapsed MV segments, n (%)	
A1 prolapse	11 (7.1)
A2 prolapse	38 (24.7)
A3 prolapse	26 (16.9)
P1 prolapse	16 (10.4)
P2 prolapse	78 (50.6)
P3 prolapse	47 (30.5)
Commissure prolapse	22 (14.3)
Multiple segment prolapse	40 (26.0)
Barlow disease, n (%)	11 (7.1)

*IQR*, Interquartile range; *NYHA*, New York Heart Association; *LV*, left ventricular; *TR*, tricuspid regurgitation; *MV*, mitral valve.

### Surgical Details

Details of the operation are shown in Table 2. Chordal replacement with loop technique was performed in 136 patients (88.3%), whereas leaflet resection was performed in only 1 patient (0.6%). Ninety-five patients (61.7%) underwent edge-to-edge repair of the commissural segments. The median number of neochords was 2.4 (IQR, 2-3). All the patients underwent annuloplasty ring using the Physio II semirigid ring, with a median prosthesis size of 30 mm (IQR, 28-30 mm). Surgical approaches included sternotomy in 139 patients (90.3%) and right mini-thoracotomy in 15 patients (9.7%). The median operation time, cardiopulmonary bypass time, and cross clamp time were 332 (IQR, 281-353) minutes, 176 (IQR, 137-200) minutes, and 138 (IQR, 101-157) minutes, respectively.

**Table 2:** Surgical details (N = 154)

Variable	Value
Repair technique	
Chordal replacement; loop technique, n (%)	136 (88.3)
Number of neochords, median (IQR)	2.4 (2-3)
Triangular leaflet resection, n (%)	1 (0.6)
Edge-to-edge repair, n (%)	95 (61.7)
Cleft suture, n (%)	17 (11.0)
Annuloplasty	
Semirigid ring, n (%)	154 (100)
Prosthesis size, median (IQR)	30 (28-30)
Surgical approach, n (%)	
Sternotomy	139 (90.3)
Right mini-thoracotomy	15 (9.7)
Concomitant procedures, n (%)	
Tricuspid valve repair or replacement	18 (11.7)
MAZE procedure	42 (27.3)
Coronary artery bypass grafting	12 (7.8)
Operation time, min, median (IQR)	332 (281-353)
Cardiopulmonary bypass time, min, median (IQR)	176 (137-200)
Cross-clamp time, min, median (IQR)	138 (101-157)

*IQR*, Interquartile range.

### In-Hospital Outcomes and Postoperative Echocardiography Findings

In-hospital outcomes are indicated in Table 3. There were no in-hospital deaths or conversions to MV replacement. No patients experienced postoperative stroke. Intraoperative SAM was observed in 9 patients (5.8%). Echocardiography at 2 weeks after surgery revealed 99.4% of patients with MR grade 0-1, 0.6% with grade 2%, and 0% with grade 3-4. The mean transmitral pressure gradient (MTPG) was 3.1 mm Hg (IQR, 2-4 mm Hg). Seventeen patients (12.9%) had an MTPG ≥5 mm Hg. Regarding the morphology of the MV after repair, the median coaptation length, anterior leaflet coaptation length, and posterior leaflet coaptation length were 10.0 mm (IQR, 8.4-11.5 mm), 10.7 mm (IQR, 8.8-12.2 mm), and 12.3 mm (IQR, 9.7-14.6 mm). The median coaptation depth was 4.8 mm (IQR, 3.7-5.9 mm), and the median AP diameter was 21 mm (IQR, 19.1-22.8 mm).

**Table 3:** In-hospital outcomes and postoperative echocardiography findings (N = 154)

Variable	Value
In-hospital death or 30-d mortality, n (%)	0 (0)
Conversion to mitral valve replacement, n (%)	0 (0)
Intensive care unit length of stay, d, median (IQR)	3.0 (2-3)
Hospital length of stay, d, median (IQR)	33 (21-30)
Complications, n (%)	
Mechanical circulatory support	0 (0)
Myocardial infarction	0 (0)
Aortic dissection	0 (0)
Stroke	0 (0)
Respiratory failure	4 (2.6)
Renal failure	4 (2.6)
Deep surgical site infection	1 (0.6)
Postoperative echocardiography findings	
LV diastolic diameter, mm, median (IQR)	47 (42-51)
LV systolic diameter, mm, median (IQR)	33 (28-37)
Left atrial diameter, mm, median (IQR)	38 (33-43)
LV ejection fraction, %, median (IQR)	53 (50-59)
MV regurgitation grade, n (%)	
Grade 0-1	153 (99.4)
Grade 2	1 (0.6)
Grade 3-4	0 (0)
Systolic anterior motion, n (%)	9 (5.8)
Mean transmitral pressure gradient, mm Hg, median (IQR)	3.1 (2-4)
Transmitral pressure gradient >5 mm Hg, n (%)	17 (12.9)
Morphology of MV after repair, median (IQR)	
Coaptation length, mm	10.0 (8.4-11.5)
Anterior leaflet coaptation length, mm	10.7 (8.8-12.2)
Posterior leaflet coaptation length, mm	12.3 (9.7-14.6)
Coaptation depth, mm	4.8 (3.7-5.9)
AP diameter, mm	21.0 (19.1-22.8)
Anterior leaflet length in the coaptation triangle, mm	17.5 (15.1-20.0)
Posterior leaflet length in the coaptation triangle, mm	5.8 (4.7-7.2)
Anterior leaflet angle, °	19.0 (13.2-23.2)
Posterior leaflet angle, °	72.9 (50.7-91.0)
Coaptation point-septal length, mm	25.5 (21.1-29.5)

*IQR*, Interquartile range; *LV*, left ventricular; *MV*, mitral valve; *AP*, anterior-posterior.

### Comparison of MV Morphology After MVr

**Figure 2:**
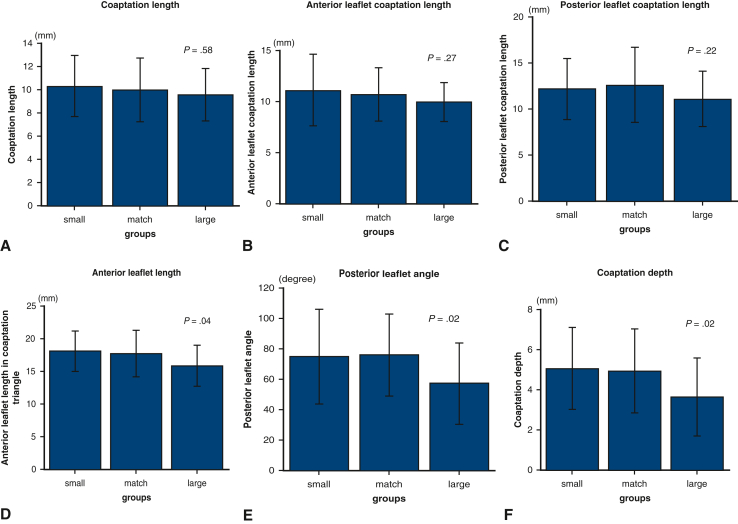
The upper border of the box represents the mean value, and the whiskers in the box show the range of the standard deviation. A, The median coaptation length of the small, match, and large groups was 10.3 mm, 10.0 mm, and 9.5 mm, respectively, with no significant difference among 3 groups (*P* = .58). B, The median anterior leaflet coaptation lengths were 11.1 mm, 10.7 mm, and 10.0 mm, respectively, with no significant difference among the 3 groups (*P* = .27). C, The median posterior leaflet coaptation lengths were 12.2 mm, 12.7 mm, and 11.1 mm, respectively, with no significant difference among the 3 groups (*P* = .22). D-F, The patients in the large group had significantly shorter median anterior leaflet length (18.1 mm, 17.7 mm, and 15.9 mm, respectively; *P* = .04) (D), smaller median posterior leaflet angle (74.6°, 75.9°, and 57.1°, respectively; *P* = .02) (E), and shorter median coaptation depth (4.9 mm, 5.0 mm, and 3.6 mm, respectively; *P* = .03) (F).

The study cohort included 40 patients in the small group, 92 in the match group, and 22 in the large group, based on the actual ring size compared to the preoperatively predicted ring size. The median coaptation length of the 3 groups was 10.3 mm, 10.0 mm, and 9.5 mm, respectively (*P* = .58) (Figure 2, *A*). The median anterior leaflet coaptation length in the 3 groups was 11.1 mm, 10.7 mm, and 10.0 mm, respectively (*P* = .27) (Figure 2, *B*), whereas the median posterior leaflet coaptation length was 12.2 mm, 12.7 mm, and 11.1 mm (*P* = .22) (Figure 2, *C*). The patients in the large group had significantly shorter anterior leaflet length in the coaptation triangle (18.1 mm, 17.7 mm, and 15.9 mm, respectively; *P* = .04) (Figure 2, *D*), smaller posterior leaflet angle (74.6°, 75.9°, and 57.1°, respectively; *P* = .02) (Figure 2, *E*), and shorter coaptation depth (4.9 mm, 5.0 mm, and 3.6 mm, respectively; *P* = .03) (Figure 2, *F*).

### Long-Term Outcomes

**Figure 3:**
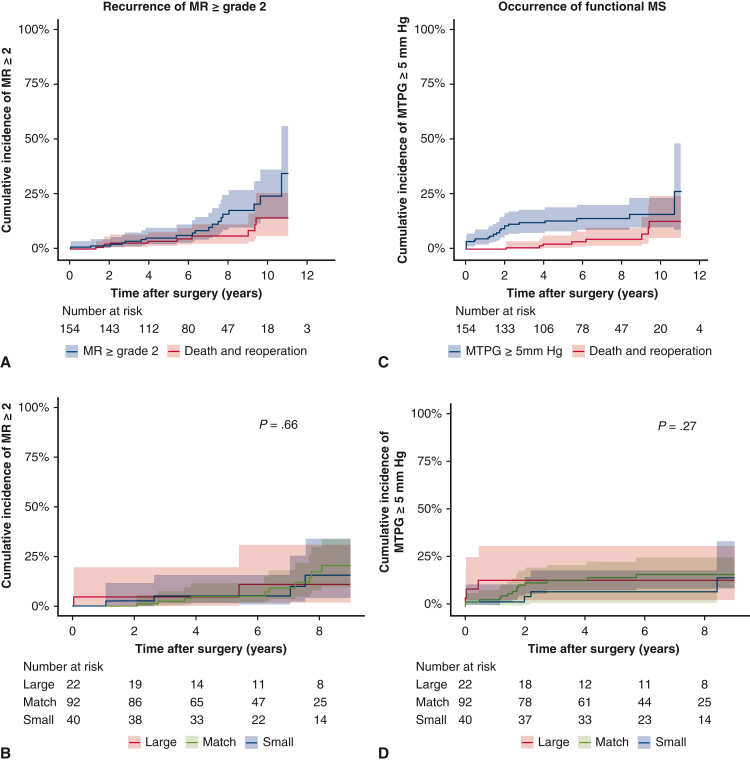
A, The 1-, 5-, and 10-year cumulative incidence of recurrent mitral regurgitation (MR) grade ≥2, with death and reoperation as competing risks, was 0.6%, 6.3%, and 24.0%, respectively. B, There was no significant difference in the rate of recurrence of MR grade ≥2 among the 3 groups (*P* = .66). C, The 1-, 5-, and 10-year cumulative incidence of MTPG ≥5 mm Hg, with death and reoperation as competing risks, was 4.6%, 13.0%, and 16.0%, respectively. D, No significant difference in the incidence of mean transmitral pressure gradient (MTPG) ≥5 mm Hg was observed across the 3 groups (*P* = .48). *Shaded areas* indicate 95% confidence intervals.

**Figure E1:**
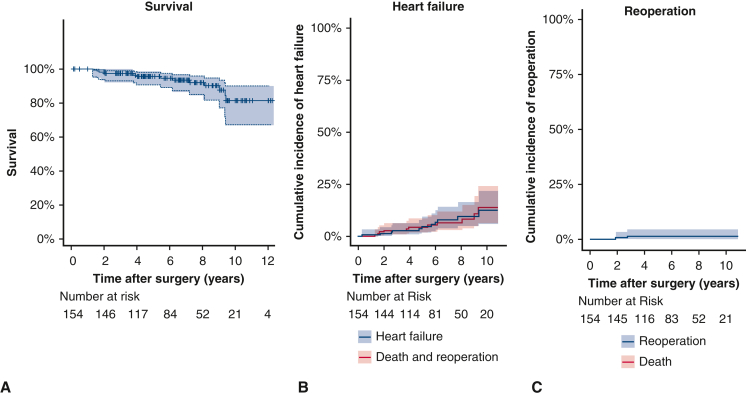
A, On Kaplan-Meier analysis, the 1-, 5-, and 10-year survival rate was 100%, 95.7%, and 81.3%, respectively. B, The 1-, 5-, and 10-year cumulative incidence of heart failure requiring readmission, with death and reoperation as competing risks, was 0.6%, 4.7%, and 12.5%, respectively. C, The 1-, 5-, and 10-year cumulative incidence of mitral valve reoperation, with a death as competing risk, was 0%, 1.4%, and 1.4%, respectively. *Shaded areas* indicate 95% confidence intervals.

Over the median follow-up of 6.4 years (IQR, 4.1-9.0 years), there were 13 deaths, caused by a malignant tumor in 3 patients, an aortic event in 2, pneumonia in 4, and unknown cause in 4. The Kaplan-Meier analysis revealed 1-, 5-, and 10-year survival rates of 100%, 95.7%, and 81.3%, respectively (Figure E1, *A*). The 1-, 5-, and 10-year cumulative incidence of heart failure requiring readmission with death and reoperation as competing risks was 0.6%, 4.7%, and 12.5%, respectively (Figure E1, *B*). Two patients underwent redo MV replacement due to recurrent severe MR. The 1-, 5-, and 10-year cumulative incidence of mitral valve reoperation with a death as competing risk was 0%, 1.4%, and 1.4%, respectively (Figure E1, *C*), and the 1-, 5-, and 10-year cumulative incidence of recurrent MR grade ≥ 2 with death and reoperation as competing risks was 0.6%, 6.3%, and 24.0%, respectively (Figure 3, *A*). There was no significant difference in the rate of recurrence of MR grade ≥2 among 3 groups (Figure 3, *B*). On the other hand, the 1-, 5-, and 10-year cumulative incidence of MTPG ≥ 5 mm Hg with death and reoperation as competing risks was 4.6%, 13.0%, and 16.0%, respectively (Figure 3, *C*). No significant difference in the incidence of MTPG ≥5 mm Hg was seen among the 3 groups (Figure 3, *D*).

### Risk Factors for Recurrent MR Grade ≥2 or Functional MS Occurrence (MTPG >5 mm Hg) by a Multivariable Fine-Grey Regression Model

Risk factors for recurrent MR grade ≥2 with death and reoperation as competing risks by a multivariable Fine-Grey regression model was larger posterior leaflet angle after MVr (hazard ratio [HR], 1.02; 95% confidence interval [CI] 1.00-1.04; *P* = .02) (Table 4). Whereas risk factors for functional MS occurrence (MTPG ≥5 mm Hg) were larger BSA (HR, 38.5; 95% CI, 3.04-487.9; *P* = .005), smaller prosthesis size (HR, 0.75; 95% CI, 0.57-0.99; *P* = .047), and shorter coaptation length (HR, 0.75; 95% CI, 0.59-0.96; *P* = .02) (Table 5).

**Table 4:** Risk factors for recurrent MV regurgitation grade ≥2 using a multivariable Fine-Grey regression model

Variables	HR	95% CI	*P* value
Age	1.02	0.97-1.06	.48
Body surface area	0.17	0.01-3.49	.25
Permanent atrial fibrillation	0.93	0.31-2.76	.90
Multiple leaflet prolapses	1.28	0.53-3.13	.59
Prosthesis			
Prosthesis size	1.11	0.87-1.41	.41
Prosthesis size mismatch	1.10	0.52-2.34	.81
Morphology of MV after repair			
Coaptation length	0.82	0.63-1.06	.13
Coaptation depth	0.95	0.69-1.30	.74
Posterior leaflet angle	1.02	1.00-1.04	.02

*HR*, Hazard ratio; *CI*, confidence interval; *MV*, mitral valve.

**Table 5:** Risk factors for mean MV pressure gradient >5 mm Hg using a multivariable Fine-Grey regression model

Variables	HR	95% CI	*P* value
Age	1.0	0.96-1.05	.92
Body surface area	38.5	3.04-487.9	.005
Permanent atrial fibrillation	0.68	0.22-2.12	.51
Multiple leaflet prolapses	2.1	0.78-5.37	.14
Prosthesis			
Prosthesis size	0.75	0.57-0.99	.047
Prosthesis size mismatch	1.56	0.63-3.85	.33
Morphology of MV after repair			
Coaptation length	0.75	0.59-0.96	.02
Coaptation depth	0.99	0.71-1.37	.93
Posterior leaflet angle	1.00	0.99-1.01	.79

*HR*, Hazard ratio; *CI*, confidence interval; *MV*, mitral valve.

## Discussion

The 10-year cumulative incidence of heart failure events necessitating hospitalization after MVr at our institution was 12.5%. Additionally, the redo rate due to MR recurrence over 10 years was 1.4%, indicating acceptable outcomes.

This study yielded the following findings. First, despite discrepancies between the predicted ring size and the actually implanted ring size, no significant differences were observed in coaptation length. It has been suggested that a smaller AP diameter ring is preferable to achieve more coaptation.^12^^,^^13^ Even when smaller-than-predicted sizes were implanted, there was no increase in coaptation length, nor was there a reduction in length when larger ring sizes were used. This may be attributed to our primary technique, the loop technique, which ensures adequate central coaptation of the MV irrespective of ring size.^14^, ^15^, ^16^ Both the anterior and posterior leaflets have surplus length beyond the coaptation area, which may help prevent the recurrence of MR even if reverse remodeling of the left ventricle occur in the long term. Additionally, the potential for implanting larger rings may contribute to the long-term prevention of functional MS.

Implanting smaller-than-predicted rings did not result in an increased incidence of SAM. This also can be attributed to the use of the loop technique on the posterior leaflet, which can pull down the posterior leaflet into the ventricle. Specifically, in our procedure, sewing the artificial chorda at the posterior leaflet 15 mm from the annulus prevents excess posterior leaflet tissue from pushing the anterior leaflet toward the left ventricular outflow tract.

**Figure E2:**
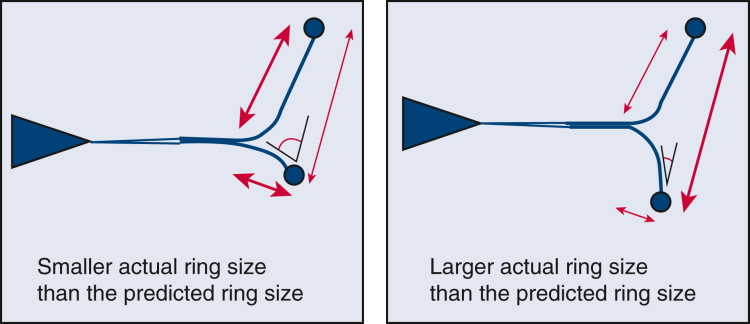
The use of larger-than-predicted ring sizes was associated with a reduction in coaptation depth, posterior leaflet angle, and anterior leaflet length within the coaptation triangle. This indicates a shift of the coaptation point from the posterior leaflet to a central position, leading to a more physiological motion of the leaflets, transitioning from a unileaflet to a bileaflet closure.

The use of larger-than-predicted ring sizes was associated with reductions in coaptation depth, posterior leaflet angle, and anterior leaflet length within the coaptation triangle. This indicates a shift in the coaptation point from the posterior leaflet to a central position, leading to a more physiologic motion of the leaflets, transitioning from a unileaflet to a bileaflet closure (Figure E2). In this study, multivariate analysis identified a larger posterior leaflet angle as an independent risk factor for MR grade ≥2. This finding suggests that unileaflet change in the MV may be associated with recurrence of MR in the long-term period; however, further investigation is warranted.

Larger BSA and smaller ring size have been identified as significant risk factors for the development of functional MS.^17^^,^^18^ However, our findings indicate that shorter coaptation length also is associated with a higher incidence of functional MS. Clinically, patients with shorter coaptation length tend to be elderly women with shorter anterior leaflets. These patients often require smaller ring sizes, which may increase the risk of functional MS. Further investigation is needed to explore this potential correlation.

This study has several limitations. First, its single-center, retrospective design may limit the generalizability of our findings. Second, the relatively small sample size might have impacted the statistical power of our analyses. Finally, the follow-up period was relatively short, and longer-term outcomes need to be investigated in future studies.

## Conflict of Interest Statement

The authors reported no conflicts of interest.

The *Journal* policy requires editors and reviewers to disclose conflicts of interest and to decline handling or reviewing manuscripts for which they may have a conflict of interest. The editors and reviewers of this article have no conflicts of interest.

## Supplementary Data

Video 1 Mitral valve repair was performed with the loop technique. The prolapsed posterior leaflet height was >15 mm, and the loop was sutured at a height of 15 mm from the annulus. Video available at: https://www.jtcvs.org/article/S2666-2736(25)00237-2/fulltext.
