# Supplementary figures and images for: Impact of the discrepancy between predicted and actual ring size on the outcomes of mitral valve repair using the loop technique
Source: JTCVS Open. 2025 Jul 21;27:46–54. doi: 10.1016/j.xjon.2025.07.008 (PMC12570550; doi:10.1016/j.xjon.2025.07.008)

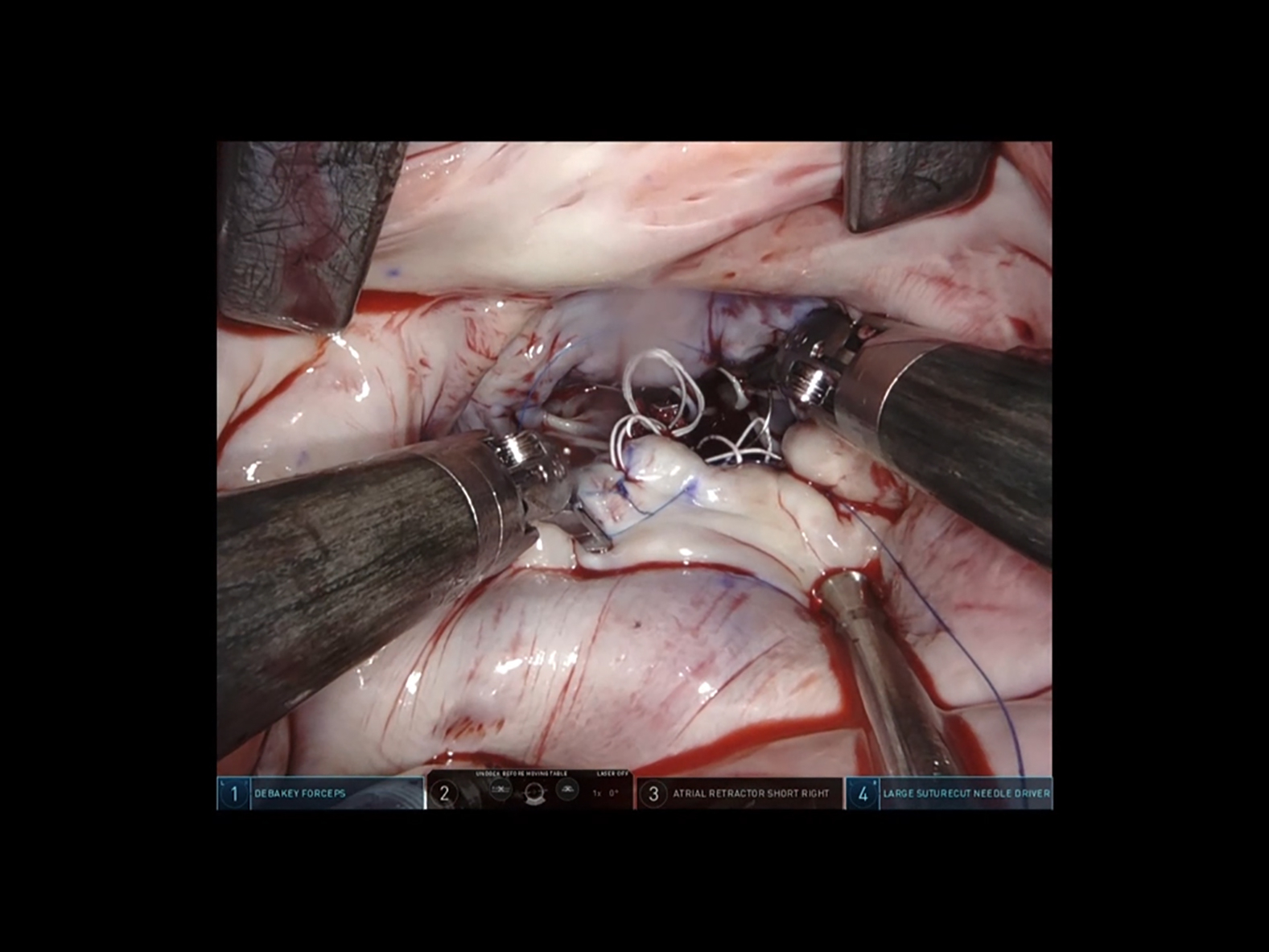

Supplement: Video 1 — Mitral valve repair was performed with the loop technique. The prolapsed posterior leaflet height was >15 mm, and the loop was sutured at a height of 15 mm from the annulus. Video available at: https://www.jtcvs.org/article/S2666-2736(25)00237-2/fulltext. [file fx2.jpg]
